# Factors associated with coverage of vitamin a supplementation among Bangladeshi children: mixed modelling approach

**DOI:** 10.1186/s12889-021-10735-7

**Published:** 2021-04-02

**Authors:** Nahyatul Marjan, Atikur Rahman, Rumana Rois, Azizur Rahman

**Affiliations:** 1 Department of Mathematics and Statistics, Bangladesh University of Business and Technology, Dhaka, Bangladesh; 2 Department of Statistics, Jahangirnagar University, Dhaka, Bangladesh; 3 School of Computing and Mathematics, Charles Sturt University, Wagga Wagga, Australia

**Keywords:** Vitamin a supplementation, Clustered data, Mixed logistic regression model, Likelihood ratio test, Intra-cluster correlation

## Abstract

**Background:**

Vitamin A deficiency (VAD) is a prominent and widespread public health problem in developing countries, including Bangladesh. About 2% of all deaths among under-five children are attributable to VAD. Evidence-based information is required to understand the influential factors to increase vitamin A supplementation (VAS) coverage and reduce VAD. We investigated the potential factors affecting VAS coverage and its significant predictors among Bangladeshi children aged 6 to 59 months using the VAS clustered data extracted from the latest Bangladesh Demographic and Health Survey 2014.

**Methods:**

Data were analysed using mixed logistic regression (MLR) modelling approach in the generalised linear mixed model framework. The MLR model performs better than logistic regression for analysing the clustered data because of its minimum Akaike information criterion value. The likelihood ratio test showed that the variance component was significant. Therefore, the clustering effect among children was inevitable to use.

**Results:**

VAS coverage among under-five children was 63.6%, which is not optimal and below the WHO’s recommendation and the country’s target of 90%. Children aged 25 to 36 months (AOR = 2.07, 95% CI: 1.711 to 2.513), who had higher educated mothers (AOR = 1.37, *p* = 0.033, 95% CI: 1.026–1.820) and fathers (AOR = 1.32, *p* = 0.027, 95% CI: 1.032–1.683), whose mothers had media exposure (AOR = 1.22, *p* = 0.006, 95% CI: 1.059–1.408) and NGO membership (AOR = 1.24, *p* = 0.002, 95% CI: 1.089–1.422) were more likely to consume VAS.

**Conclusion:**

The relevant authorities should create proactive awareness programs for highly vulnerable local communities, specifically targeted to educate the children’s mothers about the necessity and benefits of childhood nutrition.

## Introduction

Vitamin A is a fat-soluble vitamin and a promising antioxidant. It is an essential micronutrient for the proper functioning of the visual system, growth, and development, maintaining the integrity of epithelial cells, immune competence, and reproductive ability [1, 2]. Vitamin A plays a crucial role in developing strong bones, maintaining gene regulation, healthy clear skin, and facilitating cell differentiation [3]. It is also essential for child health and survival. According to a UNICEF report, globally about one-third of young children are affected [4], and around 2% of all deaths among under-five years children occurred due to vitamin A deficiency (VAD) [5]. VAD is presently considered a key symptom of preventable blindness in childhood [6]. Furthermore, VAD includes night blindness in pregnant women, which can improve the chance of maternal mortality [7].

In Bangladesh, VAD is also heavily addressed as one of the devastating health problems compared to other south Asian countries like India, Pakistan, Afghanistan, and Nepal. Nearly 44 to 55% of preschool children in South Asian countries were affected by severe VAD. In India, 62% of preschool children were reported deficient in vitamin A [8]. According to a National Nutrition Survey, 51.5% of children suffered from VAD in Pakistan [9], whereas the prevalence of VAD among children aged 6 to 59 months in Nepal is 4% [10]. In Bangladesh, a national survey, National Micronutrients Status, reported that 20.5% of preschool-age children suffer from VAD, with the highest prevalence among children living in the slums (for instance, 38% in preschool-age children and 27% in school-age children) [9]. Although the prevalence of VAD in Bangladesh in 2013 was lower than that in neighbouring country India, the prevalence of VAD remains still very high and is considered a significant public health problem in Bangladesh [9]. A child may not always be able to take vitamin A from dietary sources. The supplementation of vitamin A capsules among children is an effective technique to overcome the VAD issue in Bangladesh. During the national immunization days or campaigns on vitamin A, children aged 6 to 59 months can consume this supplement once every six months. Many developing countries have developed various programs to provide the periodic supplementation of vitamin A to increase child survival and reduce nutritional blindness incidences. VAD is high in rural areas and varied significantly with the season, ethnicity, region, and vaccination status [11].

Several studies measured the consequences of vitamin A supplementation (VAS) program and also identified the potential factors associated with low coverage of VAS in many countries. For example, parental education has been considered a potential predictor of compliance with VAS programs in Nepal and sub-Saharan African countries [7, 12–14]. The lack of maternal or caregiver education was also significantly associated with the lower performance of VAS in Nigeria [15]. In Pakistan, maternal age, living in rural areas, and regional variations were also positively associated with VAS coverage [16]. It was reported that children’s age, religious status, region, economic status, and immunization status significantly affected the lower coverage of VAS in India [17, 18]. Choi et al. [19] found that children living in poor households and whose mothers did not complete primary education were less likely to receive VAS in the Philippines. A recent study reported that the VAS program’s coverage rate gradually declines as the children grow older in Brazil [20].

In Bangladesh, one study noted that children’s age, religious status, parental education, wealth index, and breastfeeding status were significantly associated with VAS [21]. Another study reported that parents with no education and low socioeconomic status were less likely to consume VAS for their children [7, 22, 23]. Imdad et al. [24] and Mayo et al. [25] suggested that regular VAS resulted in a 24 to 28% reduction in mortality and a decrease in the incidence of night blindness. The findings on vitamin A coverage are not consistent in Bangladesh. According to the Bangladesh Demographic and Health Survey (BDHS) 2014, only 63.6% of children had consumed VAS in the preceding six months. The relevant authorities have been set a target of 90% coverage of vitamin A by 2016 through Health Population Nutrition Sector Development Program to reduce VAD prevalence [26]. Moreover, one of the most critical sustainable development goals (SDGs) is to reduce the preventable deaths of under-five year’s children at least as low as 25 per 1000 live births within 2030 [21]. Reduced VAD prevalence can achieve this essential SDG, which can be obtained by increasing the coverage of VAS consumption in children.

This study, therefore, aims to determine the potential factors for the low coverage of VAS among 6 to 59 months children in Bangladesh. Although there is much work in the literature regarding this topic, the clustering effect has been totally ignored in previous studies for analysing VAS data among Bangladeshi children [21]. Consequently, we are motivated in determining the more significant factors for the coverage of VAS by considering the regional (or cluster) effect as BDHS reported that the level of VAS coverage varied across geographical regions of the country [27]. We used VAS clustered data extracted from nationally representative BDHS 2014 [27]. In BDHS, 2014, the geographical regions were considered as a cluster with some common features. Children were selected from each cluster and hence resulting in the possible correlation among cluster responses. Discarding cluster effects can draw inaccurate inferences as well as provide a misleading interpretation of the results. In the current analysis, such clustering effect is adopted by using the mixed logistic regression modelling approach (MLR) by incorporating the random effects in addition to fixed effects via the generalised linear mixed model (GLMM) framework. This study’s findings can be of immense importance for policymakers to enable them to plan new strategies to enhance VAS coverage in Bangladesh.

## Methods

### Data sources and study design

This study used the latest survey data from BDHS, 2014 (NIPORT, 2014) to investigate the potential determinants of low coverage of VAS in Bangladesh. The survey employed a two-stage stratified random sampling design of households to collect the data. At the first stage, 600 enumeration areas (EAs) were randomly and independently selected with probability proportional to EA sizes: 207 EAs from urban and 393 EAs from rural regions. A complete list of households was then carried out with all the selected EAs used as a sampling frame for the second stage selection of households. In the second sampling stage, 30 households were chosen systematically from each EAs with an equal probability of selection. Finally, the survey interviewed 18,000 households; 6210 households were in urban areas and 11,790 in rural areas. The detailed information about the survey data is available at https://dhsprogram.com/data/available-datasets.cfm.

### Participants

Children aged 6 to 59 months were considered in this study and questioned their mother about vitamin A capsules’ consumption. Therefore, the response variable was defined by the oral statement only. Children less than six months are excluded from this study due to having breast milk. The final sample size was 6460 children information after removing all missing cases.

### Response variable

Information relating to the consumption of vitamin A capsule was collected from children’s mother through ‘is your child consume vitamin A capsule within the last six months?’. The response variable is a binary variable indicating whether a child has consumed vitamin A or not (coded as 0 = No, and 1 = Yes).

### Exposure variables

The socio-economic and demographic variables were considered as exposure variables such as children age, sex of the child, breastfeeding status, place of residence, division, religion, number of children, mother’s education, father’s education, sex of household head, wealth status, exposure of media, employment status and membership of nongovernment organisations (NGOs). For analysis, the information about selected exposure variables was not obtained directly from the survey data. The related variables from survey data were combined to compute the new variables: exposure of media and NGO membership. The variable ‘exposure of media’ was categorised as exposure if the child’s mother read newspapers or magazines or listen to the radio or watch television at least once per week. The child’s mother was also considered to be an NGO member if they belong to any one of the following NGOs: Grameen Bank, Bangladesh Rural Advancement Committee (BRAC), Bangladesh Rural Development Board (BRDB), Association of Social Advancement (ASA), Proshika, or Mother’s Club.

### Statistical models

To model the binary responses of VAS clustered data, one may conduct a logistic regression (LR) in the generalised linear model (GLM) framework. GLM violates independence assumptions to address such types of clustered data. To accommodate these clustering effects into the model, GLMM is a suitable extension of GLM that allows the possible correlation among responses [28].

Suppose *y*_*jk*_ and ***x***_*jk*_ are the binary outcome variable and *p* × 1 vector of exposure variables, respectively, collected from *k*^*th*^ (*k* = 1,  2, …, *n*_*j*_) individual of the *j*^*th*^ cluster (*j* = 1, 2, …, *l*). The correlated responses from the *j*^*th*^ cluster is denoted by ${\boldsymbol{y}}_j^T=\left({y}_{j1},\dots, {y}_{j{n}_j}\right)$, then to analyse such correlated responses, we adopted the random effects into the regression model to account for the correlation effect of the VAS data due to clustering. The MLR model is a common choice for analysing the binary response data in the context of GLMM framework. The MLR model considers the *logit* link function and can be written for the binary responses *y*_*jk*_ as

$$ g\left({\mu}_{jk}\right)= logit\ \left({\mu}_{jk}\right)=\mathit{\log}\left(\frac{\mu_{jk}}{1-{\mu}_{jk}}\right)={\boldsymbol{x}}_{jk}^{\prime }\ \boldsymbol{\beta} +{v}_j, $$

where *μ*_*jk*_ = *E*(*y*_*jk*_| *x*_*jk*_, *v*_*j*_), *g* is the *logit* link function, *v*_*j*_ is the random effect term for the *j*^*th*^ cluster that is assumed to follow a normal distribution with mean zero and variance ${\sigma}_v^2$, and ***β =*** (*β*_1_, *β*_2_, …, *β*_*p*_)^*T*^ is the *p* × 1 column vector of regression parameters [29, 30]. The estimates of the parameters ***β*** and ${\sigma}_v^2$, can be obtained by maximizing the likelihood function [28].

$$ L\left(\boldsymbol{\beta}, {\sigma}_v^2\right)=\underset{-\infty }{\overset{\infty }{\int }}\left\{\prod \limits_{j=1}^l\prod \limits_{k=1}^{n_j}f\left({y}_{jk}|{x}_{jk},{v}_j\right)\right\}d{v}_j. $$

The intra-cluster correlation coefficient (*ρ*) can be estimated by using the variance component $\left({\sigma}_v^2\right)$ as [31].

$$ \rho =\frac{\sigma_v^2}{\sigma_v^2+\frac{\pi^2}{3}}\kern0.5em . $$

For estimation, *lme4* package under the R programming language has been employed. The package also provided the estimate of an intra-cluster correlation coefficient, which quantifies the degree of correlation in the individuals’ responses within the same cluster [32]. Also, for testing the variance components ${\sigma}_v^2$ in the MLR model, we adopted the likelihood ratio test (LRT) with chi-square (*χ*^2^) test statistics [33].

Akaike’s Information Criterion (AIC) is widely used as the model selection criterion and defined as [34].

$$ AIC=-2l\left(\hat{\boldsymbol{\beta}};U\right)+2p, $$

where *p* is the number of parameters to be estimated, $l\left(\hat{\boldsymbol{\beta}};U\right)$ is the log-likelihood, $\hat{\boldsymbol{\beta}}$ is the vector of estimated model parameters, and *U* is the data vector. A model with the smallest AIC value is considered the best model for analysing the VAS data. For interpreting the results, it is more convenient to use the odds ratio (OR) to examine the covariates effect on the response variable rather than the regression coefficients. The estimated OR can be calculated by exponentiating the coefficient of individual covariate *x*_*s*_, *s* = 1, …, *p* as

$$ {OR}_s={e}^{{\hat{\beta}}_s}, $$

where ${\hat{\beta}}_s$ is the *s*^*th*^ estimated regression coefficient for *s* = 1, …, *p*.

## Results

Among 6460 children aged 6 to 59 months included in this study, 63.6% of children had consumed vitamin A capsules in the preceding six months, indicating inadequate coverage. Hence, the children were unsecured about their health nutrition. The frequency and percentage distributions of socio-economic and demographic variables of children aged 6 to 59 months in Bangladesh are presented in Table 1.

**Table 1:** Frequency and percentage distributions of socio-economic and demographic variables of children aged 6 to 59 months in Bangladesh

Variables	Number of children	Percentage (%)
Children’s age (in months)		
06–12	902	14.0
13–24	1432	22.2
25–36	1436	22.2
37–48	1416	21.9
49–59	1274	19.7
Sex of child		
Male	3319	51.4
Female	3141	48.6
Breastfeeding status		
No	2880	44.6
Yes	3580	55.4
Place of residence		
Rural	4409	68.3
Urban	2051	31.7
Division		
Dhaka	1130	17.5
Barisal	733	11.3
Chittagong	1241	19.2
Khulna	704	10.9
Rajshahi	783	12.1
Rangpur	781	12.1
Sylhet	1088	16.8
Religion		
Muslim	5916	91.6
Non-Muslim	544	8.4
Number of children		
1	4238	65.6
2	1829	28.3
3 or higher	393	6.1
Mother’s education		
No education	1055	16.3
Primary	1814	28.1
Secondary	2952	45.7
Higher	639	9.9
Father’s education		
No education	1694	26.2
Primary	1960	30.3
Secondary	1891	29.3
Higher	915	14.2
Sex of household head		
Female	602	9.3
Male	5858	90.7
Wealth index		
Poor	2648	41.0
Middle	1245	19.3
Rich	2567	39.7
Exposure of media		
Non-exposure	3213	49.7
Exposure	3247	50.3
Employment status		
Unemployed	4766	73.8
Employed	1694	26.2
NGO membership		
No	4915	76.6
Yes	1509	23.4
Vitamin A capsule consumption		
No	2354	36.4
Yes	4106	63.6

Table 1 shows that more than 50 % (66.3%) of children aged 13 to 48 months included in this study. Of the total respondents, 51.4% of the children were male, and 48.6% were female. Most of the children (55.4%) were still breastfeeding, and 44.6% were not. 68.3% of children were selected from rural and 31.7% from urban areas. The division-wise percentage of children were 17.5, 11.3, 19.2, 12.1, 12.1, and 16.8% from Dhaka, Barisal, Chittagong, Khulna, Rajshahi, Rangpur, and Sylhet, respectively. The vast majority of participants were Muslim (91.6%), while only 8.9% of children were non-Muslim. 65.6% of the mothers had one child, and only 6.1% had three or more children.

A significant percentage (16.3%) of the children’s mothers were illiterate, i.e. having no educational attainment, and only 9.9% of the children’s mothers had higher education. Among the children’s mothers, 28.1 and 45.7% had primary and secondary education levels. Besides, 26.2% of children’s fathers were illiterate, and 14.2% had higher education. The percentage of children’s fathers having primary and the secondary education is almost the same (i.e. 30.3% had primary and 29.3% had secondary education). The vast majority (90.7%) of children reside in male-headed households and only 9.3% of children in female-headed households. The proportion of children from low-income families was higher (41.0%) than rich (39.7%) and middle class (19.3%) families. More than 50 % (50.3%) of the children’s mothers had mass-media exposure in Bangladesh. Table 1 also shows that most of the children’s mothers were unemployed (73.8%), and only 26.2% were employed. The major 76.6% of respondents’ mothers were not involved with NGOs, while the rest (23.4%) had involvement with NGO activities.

The results obtained from the bivariate analysis are presented in Table 2. Among respondents, a higher proportion (67.5%) of children aged 25 to 36 months had consumed vitamin A capsules than other aged groups. The age of Bangladeshi children was significantly associated (*p* < 0.001) with their status of VAS. An elevated proportion (66.1%) of VAS status among non-breastfeeding children were found, while the percentage was 61.5% for breastfeeding children. Breastfeeding status (p < 0.001) of children was also significantly associated with their VAS status in Bangladesh. At a 5% level of significance, the place of residence was significantly associated (*p* = 0.012) with participants’ VAS status. The regional variation among children was strongly associated (*p* < 0.001) with their VAS consumption in Bangladesh. Participants reported the highest percentage of VAS consumption among children in the Rangpur division, whereas the lowest percentage in the Sylhet region.

**Table 2:** Frequency and percentage distributions of VAS status among children aged 6 to 59 months in Bangladesh along with p-value of the chi-square (*χ*^2^) test

Variables	Vitamin A capsule consumption status		*χ*^2^ value	*P*-value
	No (%)	Yes (%)		
Children’s age (in months)				
06–12	433 (48)	469 (52.0)	66.297	< 0.001^**^
13–24	512 (35.8)	920 (64.2)		
25–36	467 (32.5)	969 (67.5)		
37–48	478 (33.8)	938 (66.2)		
49–59	464 (36.4)	810 (63.6)		
Sex of child				
Male	1238 (37.3)	2081 (62.7)	2.184	0.139
Female	1116 (35.5)	2025 (64.5)		
Breastfeeding status				
No	977 (33.9)	1903 (66.1)	14.204	< 0.001^**^
Yes	1377 (38.5)	2203 (61.5)		
Place of residence				
Rural	1652 (37.5)	2757 (62.5)	6.351	0.012^*^
Urban	702 (34.2)	1349 (65.8)		
Division				
Dhaka	402 (35.6)	728 (64.4)	31.612	< 0.001^**^
Barisal	248 (33.8)	485 (66.2)		
Chittagong	430 (34.6)	811 (65.4)		
Khulna	252 (35.8)	452 (64.2)		
Rajshahi	319 (40.7)	464 (59.3)		
Rangpur	248 (31.8)	533 (68.2)		
Sylhet	455 (41.8)	633 (58.2)		
Religion				
Muslim	2184 (36.9)	3732 (63.1)	6.907	0.009^**^
Non-Muslim	170 (31.3)	374 (68.8)		
Number of children				
1	1491 (35.2)	2747 (64.8)	8.672	0.013^*^
2	706 (38.6)	1123 (61.4)		
3 or higher	157 (39.9)	236 (60.1)		
Mother’s education				
No education	460 (43.6)	595 (56.4)	74.789	< 0.001^**^
Primary	738 (40.7)	1076 (59.3)		
Secondary	984 (33.3)	1968 (66.7)		
Higher	172 (26.9)	467 (73.1)		
Father’s education				
No education	694 (41.0)	1000 (59.0)	76.731	< 0.001^**^
Primary	800 (40.8)	1160 (59.2)		
Secondary	606 (32.0)	1285 (68.0)		
Higher	254 (27.8)	661 (72.2)		
Sex of household head				
Female	224 (37.2)	378 (62.8)	0.170	0.680
Male	2130 (36.4)	3728 (63.6)		
Wealth index				
Poor	1099 (41.5)	1549 (58.5)	52.922	< 0.001^**^
Middle	435 (34.9)	810 (65.1)		
Rich	820 (31.9)	1747 (68.1)		
Exposure of media				
Non-exposure	1324 (41.2)	1889 (58.5)	62.743	< 0.001^**^
Exposure	1030 (31.7)	2217 (68.3)		
Employment status				
Unemployed	1729 (36.3)	3037 (63.7)	0.206	0.650
Employed	625 (36.9)	1069 (63.1)		
NGO membership				
No	1859 (37.5)	3092 (62.5)	11.241	0.001^**^
Yes	495 (32.8)	1014 (67.2)		

** statistically significant at 1% level of significance, * significant at 5% level

From Table 2, it can be observed that the non-Muslim children consumed more (68.8%) VAS than Muslim children (63.1%). The religious status of children (*p* = 0.009) in Bangladesh was strongly associated with their VAS status. Though the association between the number of children of mothers and their children’s VAS status was insignificant (*p* = 0.013) at a 1% level of significance, it was significant at a 5% level of significance. It seems that mothers with fewer children were more conscious of consuming vitamin A capsules by their children. For instance, the percentage of VAS consumed children were 64.8, 61.4, and 60.1% for mothers who have one child, two children, and three or more children, respectively.

The educational attainment of both children’s mothers (*p* < 0.001) and fathers (*p* < 0.001) was strongly associated with their VAS status, which stipulates that higher educated parents were more concerned about VAS consumption to their children. The proportion of VAS consumption was observed to rise with the increasing levels of wealth index, and this index of children (*p* < 0.001) was strongly associated with the coverage of VAS in Bangladesh. VAS consumed children were significantly (*p* < 0.001) more likely than non-consumed children to be had mass-media exposure mothers (68.3%). NGO membership of children’s mother was also strongly associated (*p* = 0.001) with their status of VAS. The percentage of children consuming VAS was higher (67.2%) among mothers with NGO membership than their counterparts.

The selected exposure variables that were statistically significant with VAS coverage in bivariate analysis (Table 2), were further used in multivariate analysis to determine significant potential factors of VAS coverage among Bangladeshi children. Although the place of residence and number of children showed insignificant association (but significant at a 5% level of significance) with VAS coverage, they were included in the regression model of finding adjusted effects. We applied both LR and MLR in the context of GLMs and GLMMs framework, respectively. The results are summarised in Table 3, along with the estimated variance component $\left({\hat{\sigma}}^2\right)$, the intra-cluster correlation coefficient (*ρ*), LRT, and AIC values.

**Table 3:** Models selection criterion (AIC) for LR and MLR along with estimated variance component $\left({\hat{\sigma}}^2\right)$, intra-cluster correlation (*ρ*), chi-square (*χ*^2^) and *p*-values of the LRT

Model	${\hat{\sigma}}^2$	*ρ*	*χ*^2^	*P*-value	AIC
Logistic regression (LR)	–	–	–	–	8287.241
Mixed logistic regression (MLR)	0.347	0.095	117.854	< 0.001	**8171.387**

It was observed that the AIC value was considerably lower for the MLR (AIC = 8171.387) than LR (AIC = 8287.241) model. Besides, the values of ${\hat{\sigma}}^2$ for the random effects associated with MLR were found to be 0.347. The contribution of random effects in the MLR model was statistically significant (*p*-value< 0.001) for modelling VAS data among children in Bangladesh. More precisely, it follows that the between children’s variability obtained from different clusters was strongly associated with the lower coverage of VAS among Bangladeshi children aged 6 to 59 months. It can also be seen that the intra-cluster correlation coefficient value was 0.095 for VAS clustered data. Finally, the MLR is the most appropriate modelling technique compared to the LR for finding the factors associated with VAS coverage for this clustered data.

The results obtained from the multivariable analysis by adopting MLR to VAS clustered data are summarised in Table 4 with the *p*-values and odds ratios (OR) along with 95% confidence intervals (CIs).

**Table 4:** Unadjusted odds ratios and adjusted odds ratios (AOR) with 95% CIs, and p-values obtained from the mixed logistic regression model

Variables	Unadjusted OR (95% CI)	*p*-values	AOR (95% CI)	*p*-values
Children’s age				
06–12 (ref.)	1.00	–	1.00	–
13–24	1.766 (1.472, 2.119)	< 0.001^**^	1.75 (1.458, 2.100)	< 0.001^**^
25–36	2.063 (1.717, 2.478)	< 0.001^**^	2.07 (1.711, 2.513)	< 0.001^**^
37–48	1.969 (1.639, 2.360)	< 0.001^**^	1.99 (1.594, 2.486)	< 0.001^**^
49–59	1.742 (1.445, 2.100)	< 0.001^**^	1.78 (1.428, 2.224)	< 0.001^**^
Breastfeeding status				
No (ref.)	1.00	–	1.00	–
Yes	0.815 (0.730, 0.910)	< 0.001^**^	1.01 (0.862, 1.185)	0.895
Place of residence				
Rural (ref.)	1.00	–	1.00	–
Urban	1.163 (0.989, 1.367)	0.068	0.97 (0.815, 1.146)	0.693
Division				
Dhaka (ref.)	1.00	–	1.00	–
Barisal	1.101 (0.830, 1.460)	0.503	1.19 (0.898, 1.577)	0.225
Chittagong	1.080 (0.838, 1.391)	0.552	1.11 (0.866, 1.424)	0.411
Khulna	0.980 (0.743, 1.293)	0.887	0.95 (0.724, 1.253)	0.729
Rajshahi	0.839 (0.641, 1.100)	0.204	0.84 (0.647, 1.104)	0.217
Rangpur	1.222 (0.929, 1.608)	0.153	1.25 (0.952, 1.646)	0.109
Sylhet	0.790 (0.606, 1.030)	0.082	0.93 (0.712, 1.205)	0.569
Religion				
Muslim (ref.)	1.00	–	1.00	–
Non-muslim	1.211 (0.966, 1.517)	0.097	1.15 (0.917, 1.447)	0.223
Number of children				
1 (ref.)	1.00	–	1.00	–
2	0.870 (0.769, 0.985)	0.027^*^	0.91 (0.797, 1.038)	0.158
3 or higher	0.797 (0.629, 1.010)	0.060	0.85 (0.663, 1.084)	0.188
Mother’s education				
No education (ref.)	1.00	–	1.00	–
Primary	1.068 (0.94, 1.262)	0.437	1.05 (0.885, 1.251)	0.567
Secondary	1.429 (1.217, 1.678)	< 0.001^**^	1.24 (1.032, 1.493)	0.022^*^
Higher	1.864 (1.474, 2.356)	< 0.001^**^	1.37 (1.026, 1.820)	0.033^*^
Father’s education				
No education (ref.)	1.00	–	1.00	–
Primary	0.962 (0.833, 1.111)	0.598	0.90 (0.775, 1.051)	0.187
Secondary	1.384 (1.190, 1.610)	< 0.001^**^	1.17 (0.984, 1.401)	0.074
Higher	1.689 (1.393, 2.047)	< 0.001^**^	1.32 (1.032, 1.683)	0.027^*^
Wealth index				
Poor (ref.)	1.00	–	1.00	–
Middle	1.240 (1.063, 1.448)	0.006^**^	1.09 (0.923, 1.284)	0.313
Rich	1.454 (1.268, 1.668)	< 0.001^**^	1.10 (0.919, 1.323)	0.294
Exposure of media				
Non-exposure (ref.)	1.00	–	1.00	–
Exposure	1.431 (1.271, 1.611)	< 0.001^**^	1.22 (1.059, 1.408)	0.006^**^
NGO membership				
No (ref.)	1.00	–	1.00	–
Yes	1.182 (1.035, 1.351)	< 0.001^**^	1.24 (1.081, 1.422)	0.002^**^

Note: *OR* 1 for the reference category, ** significant at 1% level, * significant at 5% level

Table 4 reveals that the significant predictors of VAS consumption among preschool-age children were children’s age, educational status of mothers and fathers, mass media exposure of mothers, and NGO membership of children’s mothers. Instead, children’s age, breastfeeding status, parents’ educational status, mothers’ mass media exposure and NGO membership, and family wealth index were the significant predictors as unadjusted OR. It was observed that children’s age was highly statistically significant with the status of VAS, and the maximum receiving age group was 25 to 36 months (AOR = 2.07, 95% CI: 1.711 to 2.513) than children aged 6 to 12 months. The educational status of children’s mothers showed significant effects on the consumption of vitamin A capsules. Children who had mothers educated at primary (AOR = 1.05, 95% CI: 0.0.885 to 1.251), secondary (AOR = 1.24, 95% CI: 1.032 to 1.493), or higher (AOR = 1.37, 95% CI: 1.026 to 1.820) education levels, respectively, were more likely to consume VAS than those who had mothers with no educational attainment. Similarly, fathers with higher educational attainment had a significant (*p* = 0.027) effect on VAS coverage. To be more precise, the higher educated fathers consumed 1.32 (AOR = 1.32; 95% CI: 1.032 to 1.683) times more VAS to their children than fathers with no education.

We also observed from Table 4 that mass-media exposure (*p* = 0.006) and NGO membership (*p* = 0.002) among children’s mothers were strongly associated with VAS consumption. It was observed that the proportion of consuming VAS to their children whose mothers were exposed to media and belonging to the NGO membership group were 22.0% (AOR = 1.22, 95% CI: 1.059 to 1.408) and 24.0% (AOR = 1.24, 95% CI: 1.089 to 1.422) higher, respectively, compared to their counterparts.

## Discussion

The World Health Organization (WHO) recommends high-dose vitamin A supplementation in infants and children 6 to 59 months of age, since VAD is the leading cause of preventable childhood blindness, weakens the immune system, and increases the risk of death from common childhood illnesses like measles and diarrhoea [35]. WHO has classified VAD as a public health problem affecting about one-third of children aged 6 to 59 months in 2013, with the highest rates in sub-Saharan Africa (48%) and South Asia (44%) [4]. In Bangladesh, the undernutrition prevalence has been decreased significantly and is likely to achieve the nutrition Millennium Development Goal [36]. Moreover, the country is trying to achieve the nutrition-related Sustainable Development Goals (SDGs) targets within 2030. The country’s successful VAS program has reduced VAD; nevertheless, 20.5% of children aged 6 to 59 months old are still vitamin A deficient. However, BDHS reported that VAS coverage level varied across geographical regions of the country [27]. Therefore, this study mainly focused on finding out the potential factors associated with VAS coverage incorporating the regional effects among children aged 6 to 59 months in Bangladesh. This finding can be illustrated by adopting the MLR model because it is the most appropriate modelling technique in the clustered data analysis. We used the latest clustered VAS data extracted from BDHS, 2014.

Our study findings revealed that more than three-fifths of children aged 6 to 59 months old, the same as the finding of the BDHS report, had consumed vitamin A capsules in the preceding six months, indicating inadequate coverage as it is not reaching its target of 90% coverage to achieve MDGs. Therefore, evidence-based knowledge is required to understand the factors associated with the consumption of VAS to expand its coverage. We found that children’s age, breastfeeding status, residence, division, religion, family wealth index, mother’s number of children, mother’s education, mother’s exposure of media, mother’s NGO membership, and father’s education were the significant factors associated with the consumption of VAS. VAS consumed children were more likely than non-consumed children to be aged 25 to 36 months, not in breastfeeding status, an urban resident, living in Rangpur division, non-Muslim, had mothers with only one child, had higher educated parents, from the rich-class family, had mother’s exposure of the media, had mothers with NGO membership.

The mixed logistic regression further revealed that the associated significant predictors of VAS consumption among preschool-age children are children’s age, educational status of both mothers and fathers, mass media exposure, and NGO membership of children’s mothers. The present study showed that older children were more likely to consume VAS than their younger peers in Bangladesh, whereas Agrawal et al. [17] and Lima et al. [20] reported a steady decline in the VAS coverage rate with the increasing age of children. This is probably due to a lack of mothers’ knowledge about the health benefits of vitamin A in younger children, or even they do not know the necessity of VAS during breastfeeding time to their children. Moreover, father’s and mother’s educational status were also the potential determinants of VAS coverage among children. Notably, we observed that the higher educated parents were more concerned about VAS consumption to their children. This finding is similar to many other studies that illiterate parents fail to understand the significant health consequences of VAS, and thus they are less motivated to participate in the supplementation program [7, 12, 21]. It was reported that children with illiterate mothers were at higher risk of not consuming VAS [17]. A study conducted in Cambodia found a significant association of low maternal education with lower VAS attendance, although paternal education was not associated [13]. There is sufficient literature to suggest that a lack of parental education is a crucial barrier to VAS coverage.

Our study also found that mothers’ mass media exposure and NGO membership were also significantly associated with VAS coverage. Mothers’ awareness of the consumption of VAS to their children from mass media was more likely to receive VAS. Therefore, the medias’ role could be another reason to increase the VAS coverage among children and their health care awareness. Children with NGO membership mothers were more likely to receive VAS than children whose mothers had no NGO membership. An NGO membership of mothers indicates better access to health care centres and receiving improved health care support from Bangladesh’s health system. As a result, an increase in VAS coverage. However, wealth index and religious status were not significant predictors of VAS consumption in the current study, though previous studies found them important [21, 22].

Our study has also shown that the mother’s educational status, NGO membership, and mass-media exposure had a significant role in the receipt of VAS among children aged 6 to 59 months. A plausible explanation for this finding is that mothers’ formal education, NGO membership, and mass-media exposure increases health and nutritional awareness, implies increasing awareness of VAS’s benefits. Children in the age group 6 to 12 months and breastfeeding status had a low likelihood of VAS consumption. This might be explained by the fact that mothers’ inadequate knowledge of VAS’s advantage to children health outcomes. Fathers’ formal education also increases access to information about the advantages of VAS; consequently, uptake improves of VAS. Predominantly, as the primary carer of children in Bangladesh, when mothers would be fully conscious of childhood blindness due to VAD, they may be stimulated and can become a valuable campaigner of such programs to meet the MDGs. Finally, future research may examine the social and demographic differentials of maternal factors, including health and education, and their impacts on childhood nutritional outcomes such as VAS, malnutrition, morbidity, and mortality using advanced methodologies and data science techniques [37–39].

## Conclusion

VAS coverage among children aged 6 to 59 months based on the national surveyed data was 63.6%, which is not optimal and below the WHO’s recommendation and country’s target of 90%. Based on the study findings, we recommend that government and community programs focus on creating awareness among the children’s mothers in local communities, so they can be conscious about morbidity, mortality, and blindness due to VAD in Bangladesh and become an active promoter of such programs. Finally, women’s education also needs to be assigned top priority to increase VAS coverage among children in Bangladesh.

## Data Availability

This study used secondary data from the Bangladesh Demographic and Health Surveys (BDHS) Program. The data are available online at https://dhsprogram.com/data/available-datasets.cfm.
